# Thicker Ice Improves the Integrity and Angular Distribution of CDC48A Hexamers on Cryo-EM Grids

**DOI:** 10.3389/fmolb.2022.890390

**Published:** 2022-06-17

**Authors:** Brandon Huntington, Lingyun Zhao, Patrick Bron, Umar F. Shahul Hameed, Stefan T. Arold, Bilal M. Qureshi

**Affiliations:** ^1^ Bioscience Program, Biological and Environmental Science and Engineering Division, King Abdullah University of Science and Technology (KAUST), Thuwal, Saudi Arabia; ^2^ Computational Biology Research Center, King Abdullah University of Science and Technology, Thuwal, Saudi Arabia; ^3^ Imaging and Characterization Core Lab, King Abdullah University of Science and Technology, Thuwal, Saudi Arabia; ^4^ Centre de Biologie Structurale (CBS), INSERM, CNRS, Université de Montpellier, Montpellier, France; ^5^ Division of Structural Biology (Strubi), University of Oxford, Oxford, United Kingdom; ^6^ Scientific Center of Optical and Electron Microscopy (ScopeM), ETH Zurich, Zurich, Switzerland

**Keywords:** cryo-electron microscopy, ice thickness, optimization, preferential orientation, grid preparation, particle integrity, oligomer disassembly, single particle analysis

## Abstract

Many cryogenic electron microscopy (cryo-EM) single particle analyses are constrained by the sample preparation step upon which aggregation, dissociation, and/or preferential orientation of particles can be introduced. Here, we report how we solved these problems in the case of CDC48A, a hexameric AAA ATPase from *Arabidopsis thaliana*. CDC48A hexamers are well preserved under negative staining conditions but disassemble during grid freezing using the classical blotting method. Vitrification of grids using the blot-free Chameleon method preserved the integrity of particles but resulted in their strong preferential orientation. We then used a strategy where we improved in parallel the purification of CDC48A and the conditions for cryo-EM data acquisition. Indeed, we noted that images taken from thicker ice presented an even distribution of intact particles with random orientations, but resulted in a lower image resolution. Consequently, in our case, distribution, orientation, image resolution, and the integrity of particles were tightly correlated with ice thickness. By combining the more homogeneous and stable CDC48A hexamers resulting from our improved purification protocol with an iterative search across different ice thicknesses, we identified an intermediate thickness that retained sufficiently high-resolution structural information while maintaining a complete distribution of particle orientations. Our approach may provide a simple, fast, and generally applicable strategy to record data of sufficient quality under standard laboratory and microscope settings. This method may be of particular value when time and resources are limited.

## 1 Introduction

Owing to the “resolution revolution,” cryogenic electron microscopy (cryo-EM) has become one of the major structural biology methods (Kühlbrandt, 2014). The number of structures solved by cryo-EM has risen steadily in recent years, and structures of diverse types of biological macromolecules have been successfully determined, ranging from soluble to membrane proteins, from single polypeptides to multi-domain, multi-protein, and protein-nucleic acid complexes (Nogales and Scheres, 2015; Lancey et al., 2021; Kühlbrandt, 2022). The method requires the preparation of high-quality cryo-EM grids where the sample becomes included within a thin film of vitreous ice. It is important that the ice layer contains an even and dense distribution of particles in random orientations. This sample preparation stage remains the major limiting factor for high-resolution cryo-EM analysis.

Especially multi-domain, and multi-protein complexes often require additional steps to prepare them for structural characterization. In particular, two challenges frequently occur when working with such complexes: 1) the complex is structurally heterogeneous, and/or dissociates during cryo-EM grid preparations, and 2) samples in optimally thin ice show a strong preferred orientation, and tend to dissociate or unfold at the air-water interface during grid preparation.

**FIGURE 1:**
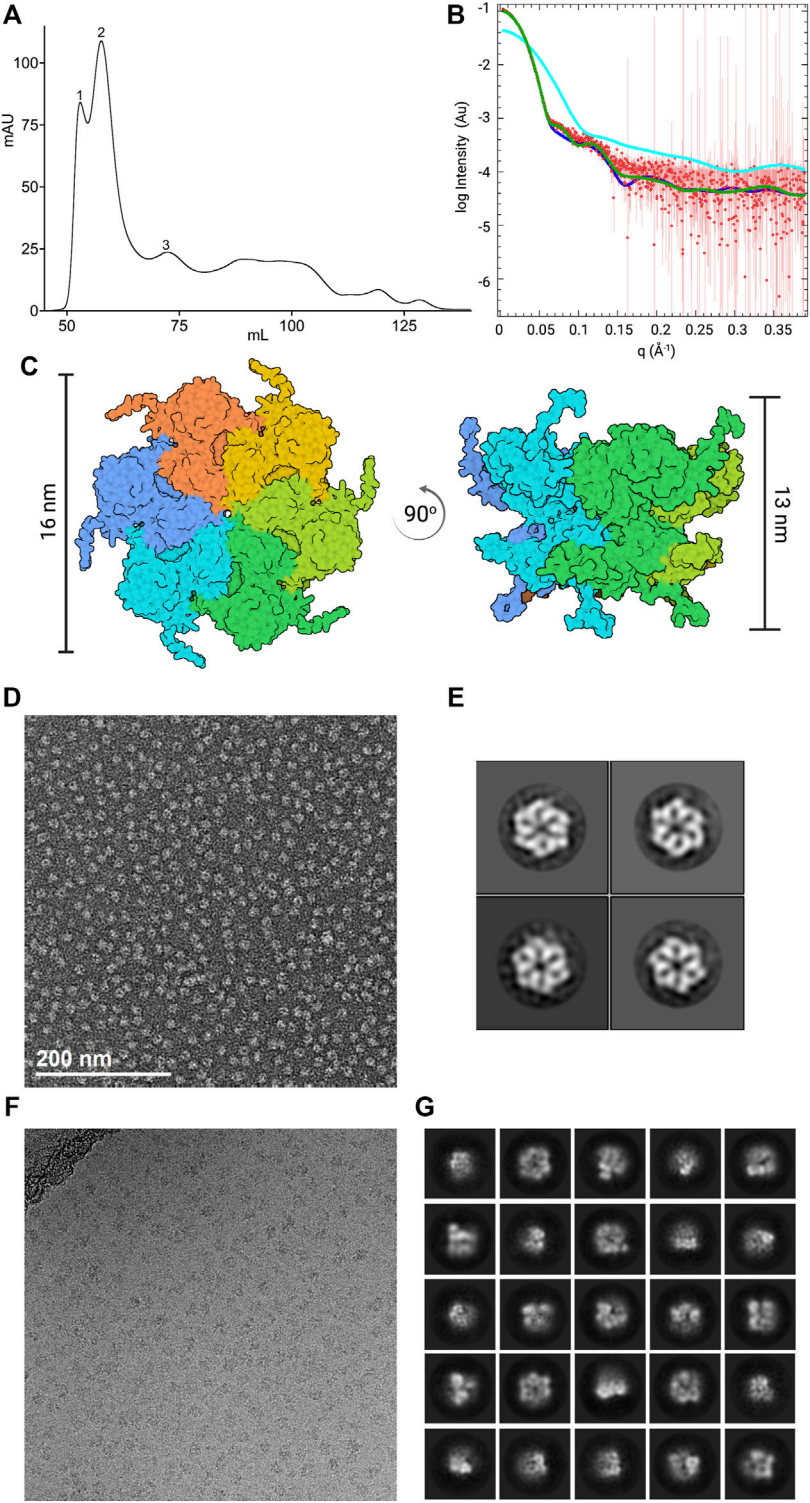
Characterization of the initial CDC48A sample. **(A)** Size-exclusion chromatography profile of CDC48A from the standard method of purification. Samples were analyzed using a HiLoad 16/600 Superdex 200 pg SEC column The trace represents absorbance at 280 nm. Based on the elution volume, the first peak corresponds to aggregates, the second and highest peak corresponds to hexameric CDC48A, and the third peak to monomeric CDC48A. **(B)** SEC-SAXS curve of CDC48A saturated with ATP (red) overlaid with SAXS patterns calculated from homology models based on the ADP-ATP state of p97 (dark blue, PDB entry 5FTN, *χ* ^2^ = 2.28), the ATP-ATP state of p97 (green, PDB entry 5FTM, *χ* ^2^ = 4.27), and an AlphaFold-derived CDC48A monomer (cyan, *χ* ^2^ = 2585). **(C)** Top view (left) and side view (right) of the best fitting model from **(B)**, colored by chain. Vertical bars represent the diameter and height, respectively. **(D)** Representative image from the NS-EM screening. **(E)** Representative 2D class averages from the NS-EM screening. **(F)** Representative cryo-EM image from the initial dataset. **(G)** Representative 2D class averages from the initial cryo-EM dataset.

Herein, we describe a simple dual strategy that resolved these challenges in our work on a flexible, multimeric nano-machine, namely the *A. thaliana* cell division cycle 48 A (CDC48A) protein complex. Being targeted to specific substrates by adaptor proteins, CDC48A extracts proteins from their cellular location, unfolds them, and routes them for degradation by the proteasome. By rapidly modifying the protein composition in specific subcellular loci, CDC48A regulates a large variety of fast-response mechanisms in plants (Liu and Li, 2014; Copeland et al., 2016; Ling et al., 2019; Huang et al., 2020). Thus, CDC48A assures a healthy proteome, and allows plants to adapt to changing conditions. CDC48A, and its homologues from fungi, and animals (where CDC48 is often called p97 or VCP) are multi-domain AAA-type ATPases that are catalytically active as a hexamer (Stach and Freemont, 2017). Each of the ∼90 kDa subunits comprises three domains, an N-terminal ligand binding domain (termed CDC48-N) followed by two ATP hydrolyzing domains (D1 and D2), and it terminates in a short flexible C-terminal tail (Figure 1C; DeLaBarre and Brunger, 2003). The molecular weight (∼550 kDa), and flexibility of the CDC48A hexamer make cryo-EM the most suitable method for its structural characterization. Cryo-EM structures of yeast, and mammalian homologues have already been reported (Banerjee et al., 2016; Cooney et al., 2019; Twomey et al., 2019; Caffrey et al., 2021; Pan et al., 2021). However, owing to a billion years of evolutionary separation, and the resulting adaptation to a very different organism, CDC48A is expected to display unique structural, and functional features. Understanding those features would provide insight into plant proteostasis, and adaptation, and may open potential biotechnological applications.

However, our efforts to structurally characterize CDC48A have been hampered by the dissociation of CDC48A hexamers during cryogenic sample preparation. Moreover, we observed large preferential orientation in optimally thin ice for high-resolution image acquisition. We herein describe how we successfully tackled these issues through a parallel approach, including optimizing sample purification, and data acquisition.

## 2 Materials and Methods

### 2.1 Protein Expression

The cDNA for CDC48A (AT3g09840) were purchased from Integrated DNA Technologies (IDT), and then subcloned to the pGEX6P-1 vector between BamHI, and NotI restriction sites. Proteins were expressed in *Escherichia coli* BL21 (DE3) cells containing an N-terminal GST tag with a PreScission protease cleavage site. The *E. coli* cells were cultured at 37°C in 2% LB Broth containing 100 µg/ml ampicillin, shaking at 200 rpm until the optical density at 600 nm reached 0.6–0.8. Subsequently, expression of CDC48A was induced using 150 µM of IPTG, and allowed to continue overnight, shaking at 200 rpm at 16°C. Cells were then pelleted by centrifugation at 7,500 rpm (12,200 × g) for 15 min at 4°C, and stored at −80°C.

### 2.2 Protein Purification

CDC48A was purified using two methodologies. The standard method is as follows: frozen cell pellets were thawed on ice, and resuspended in lysis buffer containing [50 mM Tris (pH 7.5), 200 mM NaCl, 3 mM DTT, one tablet of SigmaFast EDTA-free protease inhibitor (Sigma-Aldrich) per 50 ml preparation, and 0.5% Triton]. Cells were lysed by sonication (Branson 450 Digital Sonifier) on ice for 8 min at 35% amplitude with a pulse rate of 1 s on, and 1 s off. The lysate was pelleted by centrifugation at 27,000 rpm (87,207 × g) for 30 min at 4°C. The supernatant was added to a GST-affinity column containing glutathione sepharose beads (Cytiva) for 2 h, shaking at 4°C. The flowthrough was discarded, and the column washed with 3 column volumes (CV) of wash buffer containing [50 mM Tris (pH 7.5), 200 mM NaCl, and 3 mM DTT] followed by 1 CV of an elution buffer containing [20 mM HEPES (pH 7.5), 150 mM NaCl, and 1 mM TCEP] or [50 mM Tris (pH 7.5), 100 mM NaCl, 3 mM DTT] [for samples analyzed at Astbury Centre for Structural Molecular Biology (ACSMB), and Electron Bio-Imaging centre (eBIC), only]. CDC48A was cleaved overnight, shaking at 4°C in 15 ml of elution buffer with 3C protease. For samples analyzed at ACSMB, and eBIC, the cleavage solution additionally contained 15 units of apyrase (NEB). The eluate was then concentrated to a volume of 5 ml using an Ultra-15, 50 kDa MWCO, Amicon filtration device (Millipore), and purified using HiLoad 16/600 Superdex 200 pg (GE Healthcare) size-exclusion chromatography (SEC). Protein purity was analyzed using SDS-PAGE gels, and the relevant fractions were pooled, and stored at −80°C or used fresh, as described.

The optimized purification was carried out as above, with the following changes: one tablet of complete EDTA-containing protease inhibitor was used (Roche) per 50 ml preparation, and the cell lysate was treated with 2.5 µl of benzonase nuclease (Millipore), and incubated for 20 min at room temperature. During GST-affinity chromatography, the supernatant was added to the column for 15 min at 4°C. The flowthrough was retained, and the column washed with 4 CV of high-salt wash buffer containing [50 mM Tris (pH 7.5), 1 M NaCl, and 3 mM DTT]. The retained flowthrough was then added to the column for an additional 15 min, followed by an identical washing step. The flowthrough was discarded, and the column washed with 1 CV of elution buffer. CDC48A was similarly cleaved overnight, further purified using SEC, then stored at −80°C, or used fresh, as described.

### 2.3 Small-Angle X-Ray Scattering

SAXS data were recorded at the SWING beamline (SOLEIL, Saint-Aubin, France) using a wavelength of 1.03 Å. Scattering data were collected from a 5 mg/ml sample of CDC48A (from the standard purification after freeze-thawing once) saturated with 2 mM of ATP (Sigma-Aldrich), with the sample passed through Bio SEC-3 HPLC (Agilent) SEC prior to analysis. The distance of the sample to the detector was 1.8 m, resulting in the momentum transfer of a range of 0.1 1/Å < q < 0.5 1/Å. Buffer data were calculated from the buffer [20 mM BisTris (pH 6.5), 150 mM NaCl, 1 mM TCEP] eluted before proteins, and subtracted from the protein data using SWING’s on-site software. Data were analyzed using PRIMUS, as part of the ATSAS software package (Manalastas-Cantos et al., 2021), and models were fit to the scattering curve using FOXS (Schneidman-Duhovny et al., 2010). The monomeric CDC48A model was generated by AlphaFold (Jumper et al., 2021), and the hexameric models were generated using SWISS-MODEL (Waterhouse et al., 2018) with p97 as a template (Banerjee et al., 2016; PDB ID 5FTM, and 5FTN). Missing flexible N-terminal, and C-terminal residues were modeled using MultiProt (https://github.com/strubelab/multiprot) in several configurations, and the best fitting models were overlaid on the SAXS profile.

### 2.4 Analytical Size-Exclusion Chromatography

For comparison of the two methods of purification, an equimolar concentration of cleaved eluate after each method of purification was analyzed using Superdex 200 Increase 10/300 GL (Cytiva) SEC with the elution buffer. The absorbance at 280 and 260 nm were recorded. The protein purity of the optimized method was assessed using SDS-PAGE gels.

### 2.5 Luminescent Detection of Nucleotide

To detect the presence of nucleotides, the peak hexameric fraction (A11) from both analytical SEC experiments was analyzed using only the ADP detection step of the ADP-Glo assay (Zegzouti et al., 2009). Luminescence was detected using an Infinite M1000 Pro plate reader (Tecan) with an integration time of 1 s by preparing replicates of 10 µl CDC48A (1 mg/ml) with 20 µl of reaction buffer in 384 well plates (Corning Low Volume 384-well Black/Clear Flat Bottom).

### 2.6 EM Sample Preparation

For the initial EM experiments, CDC48A samples were prepared fresh after SEC from a fraction eluting after the hexamer peak (peak 2) to limit contamination with aggregates eluting in the neighboring peak 1 (Figure 1A). When following the optimized method, fresh sample from the center of the hexamer peak was used, unless otherwise described. Cryo-EM samples analyzed at King Abdullah University of Science, and Technology (KAUST) were prepared at 2 mg/ml (2.2 mM) with the addition of 2 mM AMP-PNP (Sigma-Aldrich). Samples analyzed at ACSMB, and eBIC had been frozen then thawed, and prepared at the desired concentration, with no additional nucleotide added.

For negative stain grid preparation, 7 µl of CDC48A (0.1 mg/ml) was placed onto glow discharged grids (Cu, 300 mesh, carbon film, EMS) for 1 min, then blotted with Whatman blotting paper. Next, ∼5 µl of 2% uranyl acetate was added to the grid, and immediately blotted. Then, ∼5 µl of 2% uranyl acetate was added again, and left to stain for 1 min. Grids were then blotted, and air-dried.

For cryogenic grid preparation at KAUST, samples were prepared using the Vitrobot Mark IV (Thermo Fisher Scientific) at 22°C with 100% humidity. CDC48A samples were prepared as previously described, applying 2 µl of sample, and blotting for 3 s. Samples from the initial CDC48A dataset, and optimized datasets 1 and 2 were prepared on glow discharged Cu grids (C-flat Cu 300 mesh R1.2/1.3, Protoships Inc.), and samples from optimized datasets 3 and 4 were prepared on glow discharged Au grids (C-flat Au 300 mesh R1.2/1.3, Protoships Inc.). For cryogenic grid preparation at ACSMB, samples were prepared using the Vitrobot Mark IV (Thermo Fisher Scientific) at 4°C in 95% humidity, with a blot time of 3 s. Grids were prepared using 2 mg/ml of CDC48A by applying 3 µl of sample to glow discharged Quantifoil Cu R1.2/1.3 grids (EMS). Cryogenic blot-free grids were prepared using the Chameleon instrument (SPT Labtech) at Rosalind Franklin Institute (RFI), United Kingdom (Razinkov et al., 2016; Dandey et al., 2018; Wei et al., 2018). Self-wicking nanowire Cu R1.2/0.8 holey carbon grids with a rectangular bar cross-section were glow discharged in a Pelco Easiglow at 12 mA, 0.39 mbar air pressure. Approximately 6 nl of the samples at 5 mg/ml were applied to each grid at a relative humidity between 75% and 85% at ambient temperature. Details, and comparison of the sample preparation for each cryo-EM dataset can be found in Supplementary Table S1.

### 2.7 EM Data Collection

Negative stain samples were screened on a Titan 80-300 (Thermo Fisher Scientific) operated at an acceleration voltage of 300 kV. Images were taken at different magnifications with a US4000 CCD camera (Gatan). For the 2D analysis of the negative stain sample, 75 images were recorded on Titan ST (Thermo Fisher Scientific) with a OneView camera (Gatan) at a magnification of 69k, and a pixel size of 1.7 Å.

Cryo-EM data were recorded in multiple facilities. At KAUST, cryo-EM data were recorded on a Titan Krios G1 (ThermoFisher Scientific) operated at 300 kV, equipped with a K2 Summit direct detector, and GIF Quantum968 Imaging Filter (Gatan company). Movies were collected at 130k magnification in super-resolution mode yielding a pixel size of 0.52 Å/pixel at specimen level. The initial dataset had 1,242 movies, optimized dataset 1 had 1,073, dataset 2 had 797, dataset 3 had 2,118, and dataset 4 had 2,284. The total exposure time was 5.6 s, dose-fractionated into 32 frames (0.175 s per frame) with a total dose of 50 e/Å^2^. At ACSMB, cryo-EM data were recorded on a Titan Krios G2 (Thermo Fisher Scientific) operated at 300 kV equipped with K2 summit direct detector, and GIF BioQuantum967 Imaging Filter (Gatan company). A total of 1,311 movies were collected at 130k magnification in electron counting mode at a pixel size of 1.07 Å/pixel. The total exposure time was 10 s, dose-fractionated into 60 frames (0.167 s per frame) with a total dose of 75 e/Å^2^. At eBIC, Diamond Light Source, cryo-EM data were recorded on a Glacios operated at 200 kV equipped Falcon IV direct detector (Thermo Fisher Scientific). A total of 1,472 movies were collected at 120k magnification in electron counting mode at a pixel size of 1.192 Å/pixel. The total exposure time was 10 s, dose-fractionated into 50 frames (0.200 s per frame), with a total dose of 48.1 e/Å^2^. A comparison of the data collection conditions across all cryo-EM datasets can be found in Supplementary Table S1.

### 2.8 EM Data Processing

Negative stain data were processed to compute 2D class averages. Briefly, the contrast transfer function (CTF) parameters were estimated by Gctf (Zhang, 2016), and particles picked automatically by Gautomatch v0.56 (https://www.mrc-lmb.cam.ac.uk/kzhang/Gautomatch/). A total of 74k particles were picked from 75 images, and extracted with a box size of 180 pixels in Relion-4.0-beta-1-commit-11e38ba (Kimanius et al., 2021), and 2D classification was performed using the VDAM algorithm.

Data collected at KAUST were motion-corrected using Warp (Tegunov and Cramer, 2019) with a binning factor of 2 and subsequently imported into cryoSPARC v3.2.0 (Punjani et al., 2017) for further processing. CTF parameters for each image were estimated by patch CTF estimation (multi). Particles were picked automatically using the Topaz wrapper in cryoSPARC (Bepler et al., 2019). Briefly, a Topaz model was trained on a curated subset of CDC48A particles selected using template picking, and subsequently used to pick the particles. Particles were extracted with a box size of 256 pixels in the initial dataset and 380 pixels in the optimized datasets. From the initial dataset, 177k particles were extracted, and subjected to further 2D classification, with no further selection of particles. From the optimized datasets, around 91k particles were extracted from dataset 1 (71k in good classes), 111k from dataset 2 (78k in good classes), 400k from dataset 3 (270k in good classes), and 550k from dataset 4 (430k in good classes). Data collected at eBIC was processed at ETH Zurich using a similar procedure as above using cryoSPARC v3.3.1. The imported movies were motion-corrected using patch motion correction (multi), and CTF parameters were estimated using patch CTF estimation (multi). Particles were picked automatically using the blob picker, and 2D class averages were computed. In a second iteration, these 2D classes were used as templates to automatically pick particles that were subsequently extracted with a box size of 350 pixels. Following 2D classification, 250k particles were retained in the good classes. Data collected at ACSMB were processed at KAUST using Relion-3.1 (Zivanov et al., 2020). Briefly, movies were subjected to motion correction using Relion’s own implementation, with no binning, and CTF parameters estimated by CTFFind-4.1 (Rohou and Grigorieff, 2015). Particles were picked automatically using Gautomatch v0.56, and 100k particles were extracted with a box size of 282, and a binning of 3. After 2D classification, 48k particles were selected and re-extracted without binning. To generate consistent figures, these particles were imported into cryoSPARC v3.3.1, and subjected to 2D classification. For each dataset, an *ab initio* reconstruction was computed using the filtered particles through the *ab Initio* Reconstruction job in cryoSPARC v3.3.1. Default reconstruction settings were used, aside from 500 extra final iterations in all, and C6 symmetry was imposed where noted. Details including the number of movies, and particles picked can be found summarized in Supplementary Table S1.

### 2.9 Ice Thickness Measurements

As an indirect measure of ice thickness, all images corresponding to each of the cryo-EM datasets were plotted according to their relative ice thickness, determined using the relative ice thickness parameter in the Manually Curate Exposure option, following patch CTF estimation (multi), in cryoSPARC v3.3.1.

As a direct measure of ice thickness, frozen aliquots of cleaved CDC48A (following the optimized methodology) were further purified using SEC. Fresh sample corresponding to the peak hexameric fraction was used to prepare cryogenic grids under the conditions used for optimized datasets 3 and 4. Ice thickness was measured using the aperture limited scattering method of Rice et al. (2018), by measuring the beam intensity of the specimen vs. the beam intensity of the vacuum (*λ* = 322 nm) on a Titan Krios operated at 300 kV using 100 micron Objective Aperture, and equipped with Gatan’s GIF/K2 detector. Ice thickness was measured from one thin ice region, one thick ice region, and two regions of intermediate ice thicknesses. For each ice thickness, five measurements were averaged.

## 3 Results

### 3.1 Quaternary Structure Disassembly During Cryogenic Sample Preparation

We recombinantly expressed *A. thaliana* CDC48A in *E. coli* and purified the protein according to our standard protocol (see *Methods*, Section 2.2). We confirmed the identity, and purity of the recombinant purified proteins through SDS-PAGE of SEC fractions (Figure 1A; Supplementary Figure S1). The SEC fractions of CDC48A that contained aggregates or monomeric protein were discarded, and only fractions from the peak containing hexamers (judged according to molecular weight standards) were retained. Additionally, SEC coupled with small-angle *x*-ray scattering (SEC-SAXS) showed that CDC48A persists as hexamers during gel filtration, and that hexamers are the prevailing species in solution, as homo-hexameric models best fitted the scattering curve (Figures 1B,C). These experiments suggested that the sample quality, and integrity was sufficient to proceed with the structural investigation.

We first performed a negative stain (NS) screening to probe the suitability of CDCD48A hexamers for cryo-EM studies. We iteratively optimized the protein concentration, and staining protocol to obtain NS-EM images of densely packed but well-separated particles (see Methods). NS-EM revealed our sample to be sufficiently monodispersed, and mostly hexameric (Figure 1D). Indeed, computing 2D class averages from the NS-EM images revealed intact hexamers of CDC48A (Figure 1E). Collectively, these analyses suggested that our sample was suitable for cryo-EM.

Next, we prepared cryogenic specimens of CDC48A at a protein concentration of 2 mg/ml (20 fold higher than used in NS) using a Vitrobot (see *Methods*), and collected the initial cryo-EM images at the Imaging, and Characterization Core Lab (IAC) in KAUST. We observed a sufficiently dense distribution of particles throughout grid regions. However, the particles did not appear homogeneous in size, and shape and differed markedly from the NS-EM images (Figure 1F). We collected a dataset of 1,242 movies on these grids and performed image processing to compute 2D class averages (Figure 1G). The 2D classes corroborated the presence of significant structural heterogeneity, apparently as a result of CDC48A hexamer dissociation. The strong dissociation of the CDC48A hexamer in cryo-EM was unexpected because intact hexamers prevailed in SEC, SAXS, and NS. The cryo-EM-specific hexamer dissociation warranted further optimization of our workflow, and protocols for the preparation of the protein sample, and cryo-EM grids.

### 3.2 Parallel Optimization Strategy

We optimized biochemical sample preparation, and cryo-EM data collection in parallel. Below, we first describe our strategy for improving the data collection (which was carried out with the protein from the initial unoptimized standard purification protocol). In the section thereafter, we report our concurrent improvements to the protein production strategy. The benefits of the improvements of grids, and samples were additive, as demonstrated in the final section that reports on the data obtained from the optimized samples with the improved data collection strategy.

#### 3.2.1 Ice Thickness Influences Complex Integrity and Particle Orientation

**FIGURE 2:**
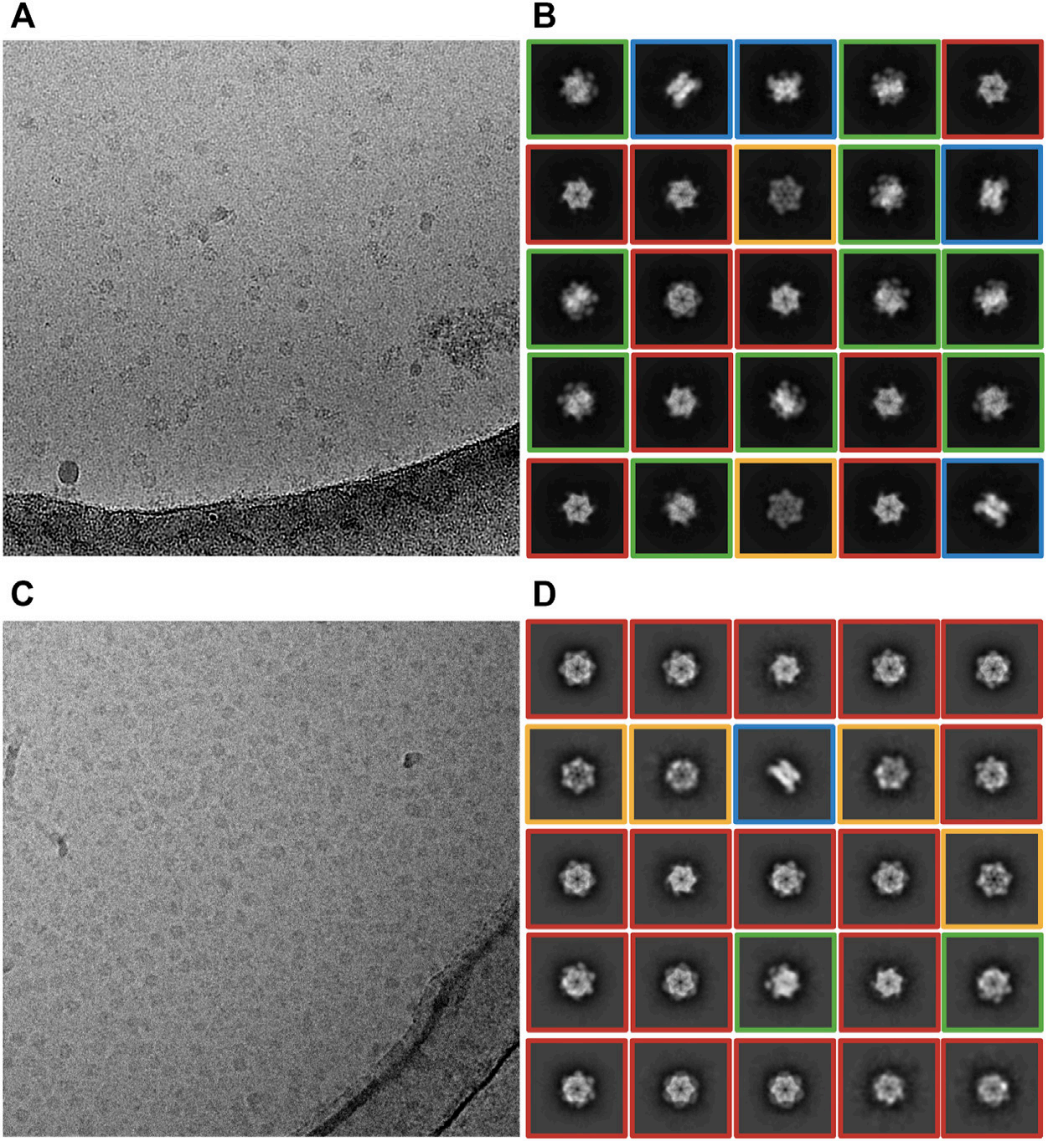
Effect of ice thickness on the distribution of particle orientations. **(A,B)** Cryo-EM data collected at ACSMB from thick ice; grids were prepared using a Vitrobot. **(A)** Representative cryo-EM micrograph. **(B)** Representative 2D class averages. Various particle orientations are noted by red boxes (top view), orange boxes (bottom view), green boxes (intermediate view) or blue boxes (bottom view). **(C,D)** Cryo-EM data collected at eBIC from optimally thin ice; Blot-free grids were prepared using the Chameleon instrument. **(C)** Representative cryo-EM images. **(D)** Representative 2D class averages. Various particle orientations are noted by red boxes (top view), orange boxes (bottom view), green boxes (intermediate view) or blue boxes (bottom view).

**FIGURE 3:**
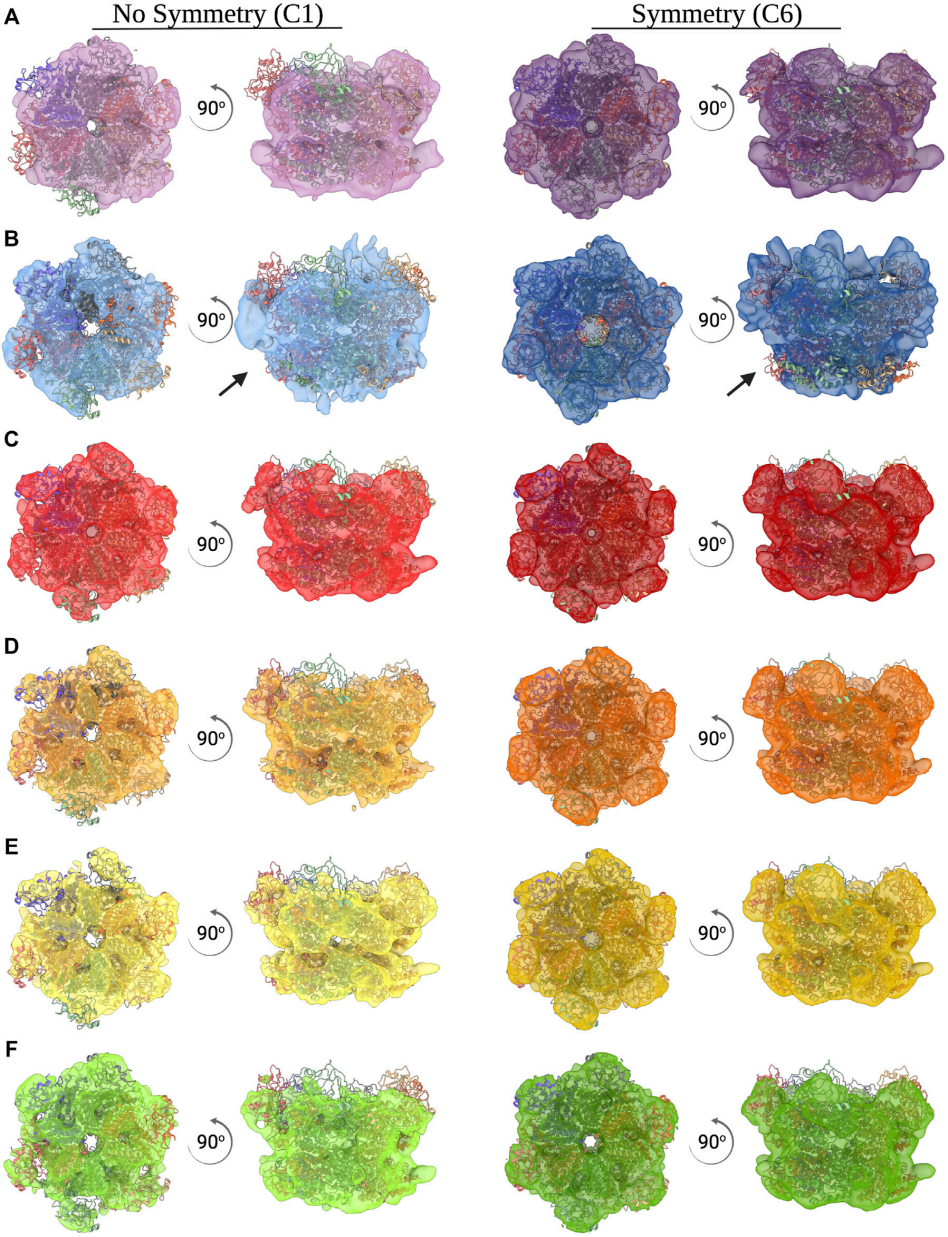
*Ab initio* reconstructions of CDC48A. **(A–F)** Top and side view of an *ab initio* reconstruction of CDC48A with (right, dark color) and without (left, light color) C6 symmetry imposed. All reconstructions from the ACSMB dataset **(A)**, the eBIC dataset **(B)**, optimized dataset 1 **(C)**, optimized dataset 2 **(D)**, optimized dataset 3 **(E)**, and optimized dataset 4 **(F)** are superimposed with the structural homologue p97 (PDB entry 5FTN). Arrows indicate poorly reconstructed regions as a result of preferential orientation.

**FIGURE 4:**
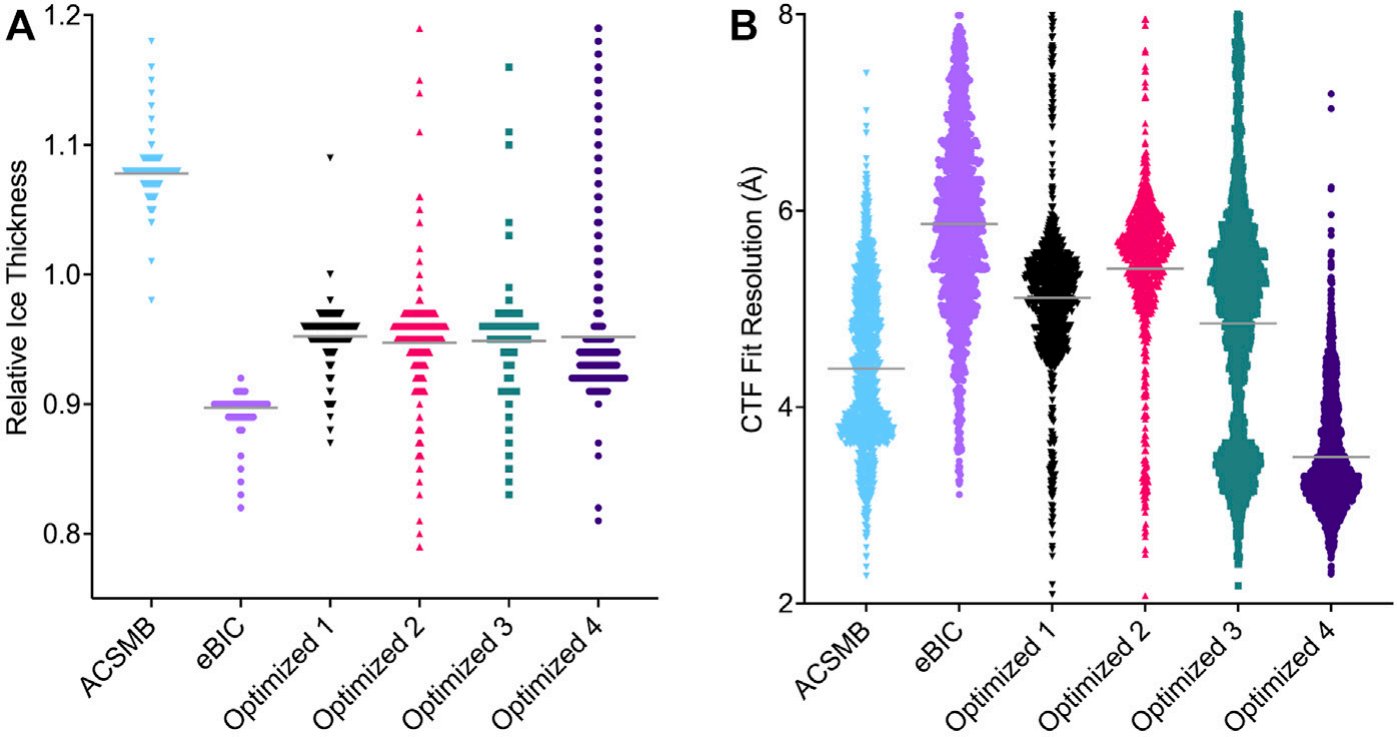
Comparison of CTF parameters estimated for each Cryo-EM dataset. Each point represents an individual image and the width of points is proportional to the number of images estimated at that value. **(A)** Estimated relative ice thickness for each image. Solid gray horizontal lines represent the mean value (ACSMB = 1.10, eBIC = 0.90, optimized dataset 1 = 0.95, 2 = 0.97, 3 = 0.97, 4 = 0.96). Images with an estimated value above 1.2 were excluded. **(B)** Estimated CTF fit resolution for each image. Solid gray horizontal lines represent the mean value (ACSMB = 4.39 Å, eBIC = 5.86 Å, optimized dataset 1 = 5.11 Å, 2 = 5.41 Å, 3 = 4.85 Å, 4 = 3.49 Å). Images with an estimated value above 8 Å were excluded.

First, we wanted to investigate whether particle dissociation was caused primarily by our handling of the sample. To this end, we cryogenically prepared grids using a slightly altered Vitrobot protocol (see *Methods*), and used them for a subsequent round of cryo-EM screening at the Astbury Centre for Structural Molecular Biology (ACSMB), University of Leeds, United Kingdom. Inspection of the images confirmed that hexamers largely dissociated in optimally thin ice regions, evidenced by an increase in background noise, and a reduction in visible hexamers. However, when screening regions of the grids that had thicker ice we noted the substantially increased prevalence of intact hexamers (Figure 2A). Collecting a dataset of 1,311 movies from thick ice and processing the data to the stage of 2D classification revealed only ∼50,000 particles. The computed 2D class averages reveal a proper distribution of particle orientations, with top and bottom views of CDC48A indicated by red and orange boxes, respectively, a few side views of CDC48A with a crab-like organization in blue and intermediate particle orientations in green (Figure 2B). The particle integrity and isotropic orientation of this dataset was also reflected in the template-free *ab initio* reconstructions with and without imposed C6 symmetry (Figure 3A). The maximum resolution of images proposed through CTF estimation in cryoSPARC ranges from 4 to 6 Å (Figure 4), indicating that these images are not suitable for computing a high resolution 3D reconstruction of CDC48A. Therefore, we concluded that capturing particles in thicker ice maintained the integrity of the hexamers, and provided a more homogeneous distribution of particle orientations.

We reasoned that the hexamer dissociation was lesser in thick ice because of a reduced surface to volume ratio, and hence a lesser exposure of proteins to the destabilizing air-water interface. To test this effect, we used the spray-on method integrated into the Chameleon grid preparation instrument (operated at eBIC, Diamond Light Source), instead of Vitrobot blotting. The faster spray-on method is expected to limit the number of interactions of the protein with the air-water interface prior to vitrification, and thus preserve the integrity of proteins (Klebl et al., 2020). Additionally, self-wicking grids are used in the Chameleon, eliminating the blotting step that may disrupt sample integrity (Wei et al., 2018). The collected dataset comprised 1,472 movies from optimal thin ice regions (Figure 2C). We extracted about 250,000 particles. The resulting 2D class averages of CDC48A revealed mainly top and bottom views of a hexameric CDC48A (Figure 2D). Thus, although the integrity of the quaternary state was maintained through the use of the Chameleon, unfortunately, only ∼2% of the extracted particles displayed the side-view orientation, resulting in a poor quality of the *ab initio* reconstruction (Table 1; Figure 3B). Therefore, thin-ice collection on Chameleon-prepared grids provided a remedy to particle dissociation but presented the problem of preferred orientation. The ice thickness of this dataset was estimated to be the thinnest of all datasets recorded. Surprisingly, the resolution of images ranged from 3 to 8 Å (Figure 4). Using the method developed by Rice et al. (2018), the holes of cryo-EM grids presenting the thinnest ice have a thickness of 21.42 ± 3.17 nm. As the diameter of CDC48A is expected to be around 16 nm, we can speculate that the shape and surface charge distribution of CDC48A, and the thickness of ice are certainly the main parameters explaining the preferential orientation of particles in this dataset. Because our access to the Chameleon was limited, we were not able to test thick-ice conditions on this instrument.

**TABLE 1:** Quantifying the preferential orientation bias. As a proxy for determining the degree of preferred orientation bias in the datasets, the number of particles that belonged to classes representing a complete side view of CDC48A was divided by the number of particles in all good classes, for each dataset. Values of 9.0 ± 1.0% correspond to an even orientational distribution.

Dataset	ACSMB	eBIC	Optimized 1	Optimized 2	Optimized 3	Optimized 4
% 90° Side view	8.40	2.00	9.60	9.70	6.40	5.20

In conclusion, using the standard Vitrobot protocol, we could either observe a dissociated complex in optimally thin ice or an intact complex in thicker ice at the cost of resolution. Conversely, blot-free vitrification by the Chameleon preserved the sample integrity but introduced a strong preferential orientation due to the asymmetrical shape of our complex that compromises its 3D reconstruction. To be able to have a rapid workflow using our in-house Vitrobot, we decided to explore optimizing the ice thickness jointly with improving the intrinsic stability of the protein sample.

#### 3.2.2 Fresh, Unconcentrated Proteins With Minimal Purification Steps Behave Best on EM Grids

**FIGURE 5:**
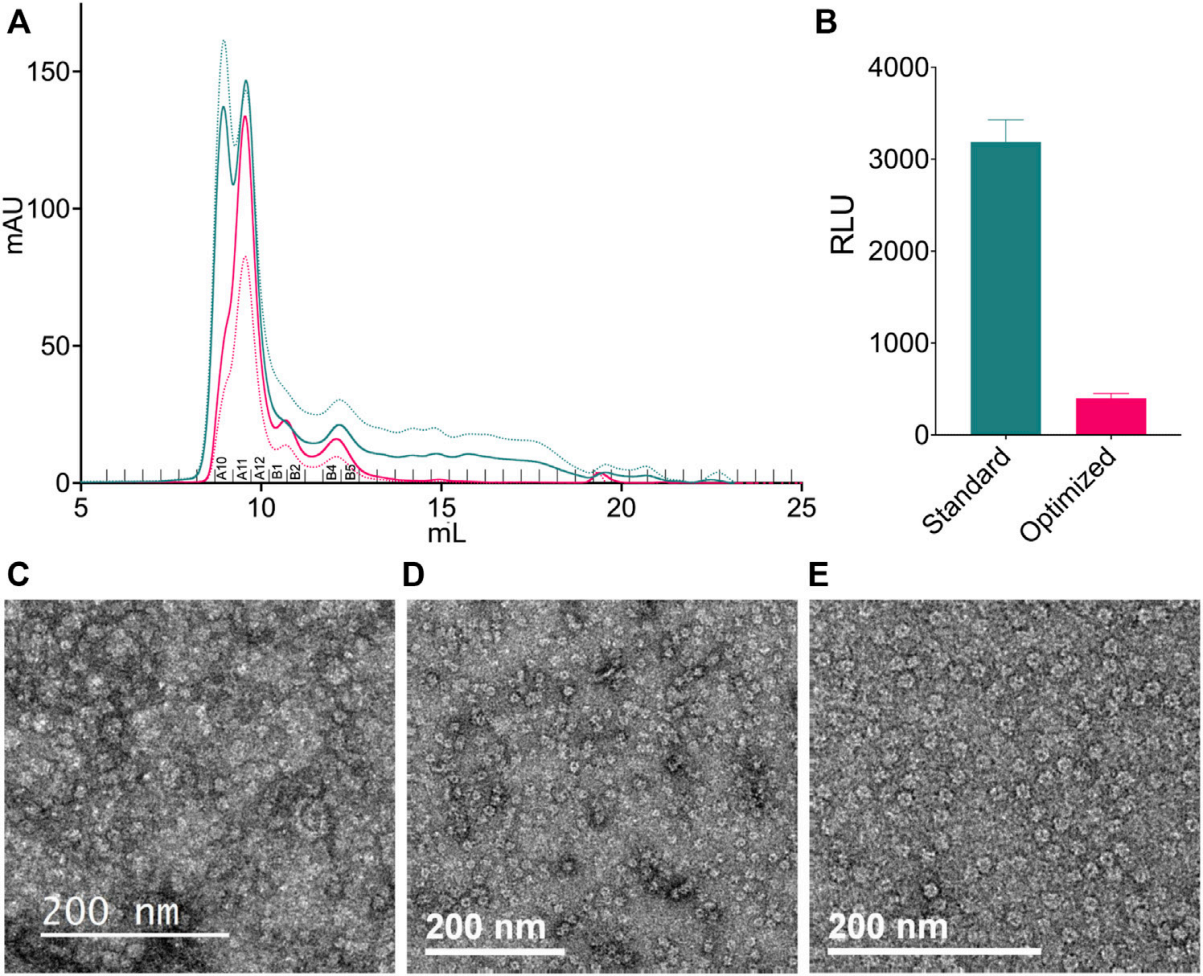
Sample preparation optimization. **(A)** Analytical SEC profiles of the standard (teal) and optimized (pink) methods of purification. Samples were analyzed using a Superdex 200 Increase 10/300 GL SEC column. Solid lines show the absorbance at 280 nm. Dotted lines show the absorbance at 260 nm. Fractions corresponding to Table 1; Supplementary Figure S1 are labeled above the *x*-axis. **(B)** Relative luminescence units (RLU) of nucleotides from fraction A11 of both purification protocols. Samples were prepared using only the detection step of the ADP-Glo assay. Error bars represent 95% confidence interval (*n* = 3). **(C,E)** NS screening of different CDC48A samples. **(C)** CDC48A samples that were thawed after pooling and concentrating for storage. **(D)** CDC48A sample taken fresh from off-center fractions of the hexameric peak (primary peak). **(E)** CDC48A sample taken fresh from the center of the hexameric peak.

To improve the stability of hexameric CDC48A complexes during grid preparation, we refined, and streamlined the purification protocol. Our standard protocol consisted of cell lysis by sonication, followed by a glutathione-S-transferase (GST) affinity column, and then a SEC. With this protocol we observed that the resulting samples displayed an absorption ratio of 260–280 nm (A260/280) of above 1 indicating strong contamination of the sample by nucleic acids and/or bound nucleotides (Table 1). Therefore, we included benzonase nuclease into the cell lysis buffer. Next, we shortened the GST-affinity column step; rather than incubating the cell lysate to bind the glutathione beads for 2 h as in the initial protocol, we incubated the lysate with the beads twice for 15 min, where each incubation step was followed by subsequent washing with 4 column volumes of a 1 M NaCl high-salt buffer (see *Methods*). This protocol quickly separated the GST-fused protein from contaminants such as proteases and reduced the overall time for the purification (Supplementary Figure S1). The high-salt washes jointly with the benzonase treatment removed interactions between our ATPase and DNA, or with other DNA-associating factors from the cell lysate. Indeed, compared with the initial purification protocol, the optimized protocol had a A260/280 ratio of ∼0.6 throughout all SEC fractions, indicative of protein alone (Figures 5A,B; Table 2). SEC also showed a substantially lower peak height for the elution volume corresponding to aggregates, suggesting a reduction of protein aggregates, possible through eliminating DNA-mediated associations (Figure 5A). Moreover, the combination of benzonase and high-salt washes also allowed us to produce CDC48A without significant amounts of bound ATP or ADP (Figure 5B). ATP is a cofactor for CDC48A, and each monomer binds two adenosine phosphate nucleotides, one each in D1 and D2 domains. The presence of ATP or ADP introduces large structural changes in p97 (Banerjee et al., 2016). Hence, the production of CDC48A molecules without this cofactor allows the preparation of homogeneous apo, ATP or ADP-loaded samples for structural and functional analysis. In conclusion, the amended purification protocol produced a purer, fresher, and more homogenous sample.

**TABLE 2:** Assessing the purity of the standard and optimized purification methods. Concentration (mg/ml) and the ratio of absorbance measured at 260–280 nm (A260/280) of fractions following analytical SEC. Fractions correspond to those labeled in Figure 5.

Fraction	Standard		Optimized	
	mg/ml	A260/280	mg/ml	A260/280
A10	1.23	1.13	0.70	0.61
A11	1.41	1.03	1.13	0.63
A12	0.69	1.14	0.65	0.66
B1	0.38	1.33	0.38	0.64
B2	0.20	1.65	0.20	0.65
B4	0.21	1.47	0.18	0.67
B5	0.17	1.60	0.10	0.57

Next, we wanted to assess the impact the new protocol had on the quality of the EM grids using NS-EM. We found that grids prepared from the peak hexameric CDC48A fractions that were pooled, concentrated, and frozen contained large amounts of aggregate clusters and indiscernible individual particles (Figure 5C). Conversely, NS-EM grids directly prepared from SEC elution fractions, without additional concentration or freezing steps, were of higher quality with more evenly distributed individual and feature-rich particles (Figures 5D,E). We also noted that grids prepared directly from the center of the peak (Figure 5E) contained particles that were more homogeneous and contained fewer aggregates compared to grids prepared from off-center peak fractions (Figure 5D). Therefore, we subsequently used only the peak fraction for the preparation of cryo-EM grids.

### 3.3 Combining an Improved Sample With Thicker Ice Yields Sufficient Data Quality

Because our initial cryo-EM work had shown disassembly and preferential orientation in thin ice for our hexameric CDC48A sample, we first investigated the behavior of our biochemically optimized protein sample in thin ice. In line with the improvements we saw in NS-EM, the optimized sample preparation increased the integrity of the sample in cryo-EM, as homogeneous and well-dispersed hexameric particles were observed under thin ice conditions following Vitrobot blotting. However, our screening of thin ice grid regions revealed a high degree of preferential orientation with particles oriented primarily in a top or bottom view. The first movies recorded from grid regions with thick ice showed a homogeneous distribution of orientations, but at the cost of high-resolution information. Therefore, we decided to start from thick ice that displays a wide distribution of particle orientations, and record datasets at different ice thicknesses to identify the optimal compromise between particle resolution, and orientation. In line with our previous observations, we did not record movies in holes presenting the thinnest ice.

Using the image grayscale as a proxy for ice thickness, we collected four separate datasets from grid regions of decreasing ice thickness and processed each dataset individually. Utilizing CTF parameter estimation in cryoSPARC, the relative ice thickness and CTF fit resolution was compared for each dataset (Figure 4). The estimated relative ice thickness obtained did not allow us to discriminate between the ice thickness of optimized datasets 1, 2 or 3, but shows clearly that dataset 4 has a thinner mean ice thickness (Figure 4A). In a second round of sample preparation using the same type of cryo-EM grids and preparation conditions, we directly measured the ice thickness according to the method of Rice et al. (2018). This indicated three different ice thicknesses for the holes used to collect the optimized datasets: 100.48 ± 6.24, 66.99 ± 8.27, and 30.60 ± 8.57 nm. The estimated CTF fit resolution revealed that datasets 1, 2, and 3 contain a majority of images with resolution ranging from 5 to 6 Å, with dataset 3 additionally containing a minority of images with resolution around 3.5 Å (Figure 4B). The estimated CTF fit resolution of the majority of images in dataset 4 range from 3 to 4 Å, indicating that this dataset is suitable to reach a final 3D reconstruction of CDC48A between 3 and 4 Å. According to our measurements, we presume that dataset 4 should have an ice thickness around 30–40 nm, while datasets 1 and 2 certainly correspond to holes having an ice thickness near 100 nm. Dataset 3 should certainly represent a mixture of images recorded in holes having ice thicknesses ranging from 30 to 100 nm, explaining why we have two populations of images within the distribution of estimated CTF fit resolution.

**FIGURE 6:**
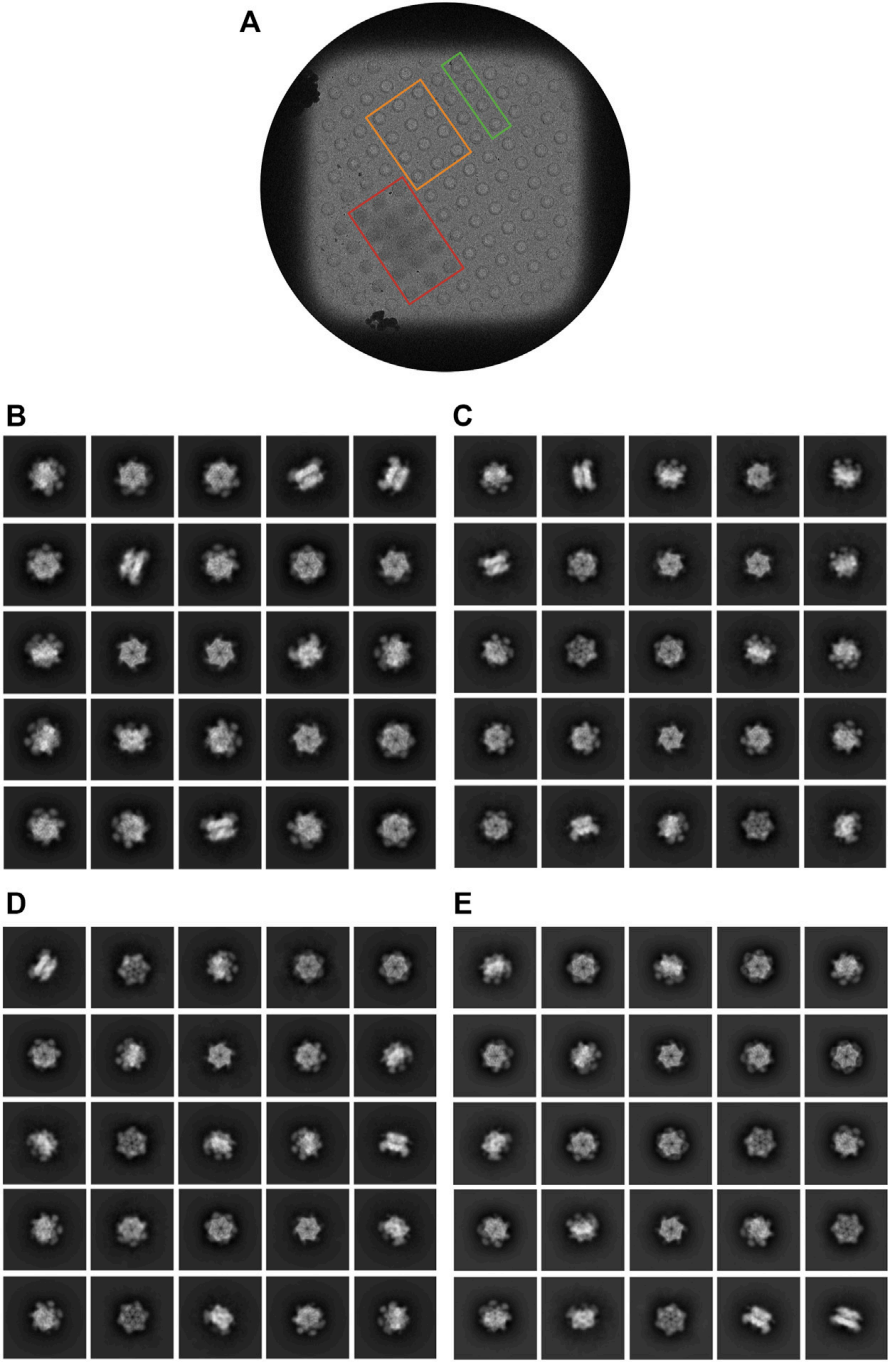
Characterization of cryo-EM data iteratively collected from regions displaying various ice thicknesses. **(A)** Representative grid square from optimized dataset 4. Examples of holes in which the ice was too thick (red), too thin (orange), or was approximately the desired thickness (green) for this dataset are outlined. **(B–E)** Representative 2D class averages from the optimized datasets 1, 2, 3, and 4, respectively.

Additionally, 2D class averages and *ab intio* reconstructions were computed for each dataset (Figure 6; Figures 3D-F). As expected from thick ice, all particle orientations were present as revealed by the top, bottom, side, and intermediate views of CDC48A seen in the 2D class average and further confirmed by the template-free *ab initio* reconstructions. The hexameric organization of CDC48A is perfectly observed in reconstructions without symmetry imposed, in which the double ring structure is evident from the side view. The reconstructions with the applied symmetry reveal a homogeneous organization of CDC48A presenting all expected structural features of CDC48A.

In conclusion, through this iterative selection process, we were able to identify dataset 4 which has an optimal ice thickness that contained both intact complexes with a sufficiently even distribution of particle orientations and sufficiently high-resolution information to allow a confident 3D structural reconstruction.

## 4 Discussion

Herein we describe our successful strategy for solving two common challenges in cryo-EM single particle analysis, namely sample integrity (in particular oligomer dissociation), and preferential particle orientation. We demonstrate that in our case, the hexameric ATPase CDC48A, these problems could be solved through the combination of efficient but non-conventional methods that led to higher oligomer stability, and orientational homogeneity.

Indeed, we initially observed that purified CDC48A displayed a hexameric organization when observed in negative stain but appeared dissociated in cryo-EM images, likely indicating that the freezing step dramatically impaired the integrity of CDC48A. It is well-described that the interaction of proteins with the air-water interface during the vitrification step can have a strong effect on the integrity of protein complexes. We showed that the Chameleon’s blot-free and spray-on method (Razinkov et al., 2016; Dandey et al., 2018; Wei et al., 2018) preserved the hexameric organization of CDC48A, confirming that dissociation of CDC48A subunits was induced during the vitification step of EM grids. In holes containing thin ice, such as those produced using this method, the thickness of the ice layer is only a few tens of nanometers. Based on the specific shape of protein complexes, and the surface charge distribution, this thickness should have an impact on the orientation of particles. In the case of CDC48A, as the hexamer is disk-like with a diameter around 16 nm, this causes particles to have a preferential orientation.

We then decided to develop a strategy where we improved in parallel the purification of CDC48A, and the conditions for cryo-EM data acquisition. One part of our strategy was to improve the workflow from cell lysis to grid preparation. Simple improvements of the handling speed, buffers, and protocols sufficiently enhanced the protein stability to enable data collection without particle dissociation, and that from ice thickness suitable for high resolution acquisition. In our case study we achieved both a higher purity and conformational homogeneity of CDC48A by removing DNA, and nucleotides from our sample. Other means for removing DNA contamination exist but may need to be assessed for unwanted side effects. For example, polyethyleneimine (PEI) treatment may lead to a significant “collateral” precipitation of the protein of interest.

A second part of our strategy was the selection of the most suitable ice thickness. It is not unexpected that thicker ice may improve the integrity of particles and their angular distribution on cryo grids. However, these effects of ice thickness are normally not systematically assessed. Moreover, in most standard settings, obtaining high-resolution data is the main focus, leading to an automated recording of movies in grid regions presenting the thinnest ice layers. In our case, we show that ice thickness correlates positively with the particle orientational coverage (Table 1), and with suitable resolution for computing atomic resolution structures of protein complexes (Figure 4). Through a simple iterative screening coupled with the analysis of 2D class averages, it was possible to identify an intermediate ice thickness that yielded both a sufficiently homogeneous particle orientation distribution and sufficiently high-resolution images (assessed by having a substantial number of images with a CTF fit resolution in the range of 3–4 Å). In our case, the method of hole selection was manual and involved visual inspection of the ice thickness or its “grayness” in images. However, it should be possible to automate screening holes according to a specific range of this parameter, as implemented via scripting in Gatan’s DM software (Rheinberger et al., 2021). Given that the movie recording speed is steadily increasing, a second option would be a “shoot first, ask questions later” approach, where data is recorded on holes of varying ice thickness, and the most appropriate ice thickness is automatically selected using analysis software such as IceBreaker (Olek et al., 2022). Ice thickness-based filtering/selection of micrographs is also implemented in cryoSPARC through the “Curate Exposures” job (Punjani et al., 2017). Although the increased ice thickness reduced the maximal resolution, the achievable resolution was, in our case, suitable for an in-depth structural, and molecular analysis (to be published later). Owing to the substantial advances of AI-based structure prediction algorithms in 2021 (Baek et al., 2021; Jumper et al., 2021), we argue that the resolution limit for addressing a functional, and biological question with cryo-EM can be lower without loss of information when confidently predicted domain structures can replace density-based *ab initio* structure modeling.

There are, of course, also other, more sophisticated ways of achieving a more homogeneous particle orientation (Drulyte et al., 2018). In our case, we could have combined the Chameleon spray-on method (Razinkov et al., 2016; Dandey et al., 2018; Wei et al., 2018), which preserved the hexameric state in thin ice, with recording a single-particle dataset on a tilted stage (Tan et al., 2017). However, access to the Chameleon is still limited for most groups. Moreover, tilting holes with thin ice will also increase the apparent ice thickness for the electrons, leading to a similar drop in maximum resolution (Drulyte et al., 2018). Additionally, setting up measurements with a tilted stage requires additional recording sessions with adjusted parameters (such as total dose), an adapted data processing workflow, and a specific instrument handling expertise that may not be available to all users (Naydenova and Russo, 2017; Tan et al., 2017).

Several other biochemical solutions have been reported to increase the particle orientation coverage in optimally thin ice, such as the use of detergents, biotinylation, antibodies, or DNA origami, in varying the pH and/or the ionic strength (Carragher et al., 2019; Han et al., 2020; Wang et al., 2020; Aissaoui et al., 2021; Patel et al., 2021). But the screening of all these parameters are extremely time consuming. In some cases, non-standard grids with a carbon or graphene support film have reduced the bias in particle orientation (Drulyte et al., 2018). However, if the anisotropy results from the particle’s shape and surface charge, it is likely that a particle that shows preferential orientation in NS-EM also does so on grids with hydrophobic support films.

In summary, although more sophisticated alternative methods exist to increase particle stability, integrity, and orientation, our parallel approach has the advantage of not requiring specific chemicals, expertise, or instruments, being based on off-the-shelf ingredients and standard procedures (chemicals, Vitroblot, untilted stage). Hence, our approach provides a robust and fast method applicable to all standard laboratory settings and cryo-EM facilities. Although our dual strategy was only tested on one project, the method is general enough to apply to other non-covalently linked oligomeric samples, samples with DNA contamination, and/or samples that show a preferential orientation in thin ice. In particular, our approach can help “salvage” grids for achieving sufficient data quality without further delays. Indeed, speed may have become a more important parameter than maximum resolution in the highly competitive cryo-EM field.

## Data Availability

The raw data supporting the conclusion of this article will be made available by the authors, without undue reservation.
