# Supplementary material for: Thicker Ice Improves the Integrity and Angular Distribution of CDC48A Hexamers on Cryo-EM Grids
Source: Front Mol Biosci. 2022 Jun 17;9:890390. doi: 10.3389/fmolb.2022.890390 (PMC9247313; doi:10.3389/fmolb.2022.890390)
Supplement: Supplementary file 1 [file DataSheet1.pdf]

Supplementary Material

1 Supplementary Tables

Supplementary Table S1. Details of each Cryo-EM dataset.

Sample Conditions	Initial Dataset	ACSMB	eBIC	Optimized 1	Optimized 2	Optimized 3	Optimized 4
**Purification method**	Standard	Standard	Standard	Optimized	Optimized	Optimized	Optimized
**Storage conditions**	Fresh	Frozen	Frozen	Fresh	Fresh	Fresh	Fresh
**Nucleotide added?**	Yes	No	No	Yes	Yes	Yes	Yes
**SEC hexameric fraction**	Off-center	Pooled	Pooled	Center	Center	Center	Center
Grid Conditions							
**Grid Type**	C-flat Cu 300 mesh R1.2/1.3	Quantifoil Cu R1.2/1.3	Self-wicking nanowire Cu R1.2/0.8	C-flat Cu 300 mesh R1.2/1.3	C-flat Cu 300 mesh R1.2/1.3	C-flat Au 300 R1.2/1.3	C-flat Au 300 R1.2/1.3
**Glow discharged?**	Yes	Yes	Yes	Yes	Yes	Yes	Yes
**Sample concentration**	2 mg/mL	2 mg/mL	5 mg/mL	2 mg/mL	2 mg/mL	2 mg/mL	2 mg/mL
**Sample volume**	2 µL	3 µL	~6 nL	2 µL	2 µL	2 µL	2 µL
**Equipment**	Vitrobot Mark IV	Vitrobot Mark IV	Chameleon	Vitrobot Mark IV	Vitrobot Mark IV	Vitrobot Mark IV	Vitrobot Mark IV

**Blot time**	3 s	3 s	-	3 s	3 s	3 s	3 s
**Temperature**	Ambient	4 ºC	Ambient	Ambient	Ambient	Ambient	Ambient
**Humidity**	100%	95%	75% - 85%	100%	100%	100%	100%
<u>Data Collection</u>							
**Collection Facility**	KAUST I&C Core Lab	University of Leeds, ACSMB	Diamond Light Source, eBIC	KAUST I&C Core Lab	KAUST I&C Core Lab	KAUST I&C Core Lab	KAUST I&C Core Lab
**Microscope**	Titan Krios G1	Titan Krios G2	Glacios	Titan Krios G1	Titan Krios G1	Titan Krios G1	Titan Krios G1
**Operating voltage**	300 kV	300 kV	200 kV	300 kV	300 kV	300 kV	300 kV
**Energy filter**	GIF Quantum968 Imaging Filter	GIF BioQuantum 967 Imaging Filter	-	GIF Quantum968 Imaging Filter	GIF Quantum968 Imaging Filter	GIF Quantum968 Imaging Filter	GIF Quantum968 Imaging Filter
**Detector**	K2 summit	K2 summit	Falcon IV	K2 summit	K2 summit	K2 summit	K2 summit
**Magnification**	130k	130k	120k	130k	130k	130k	130k
**Pixel size**	0.52 Å/pixel	1.07 Å/pixel	1.192 Å/pixel	0.52 Å/pixel	0.52 Å/pixel	0.52 Å/pixel	0.52 Å/pixel
**Frames**	32	60	50	32	32	32	32
**Total dose**	50 e/Å^2	75 e/Å^2	48.1 e/Å^2	50 e/Å^2	50 e/Å^2	50 e/Å^2	50 e/Å^2
**Exposure time**	5.6 s	10 s	10 s	5.6 s	5.6 s	5.6 s	5.6 s

Dataset Details							
**Movies**	1242	1311	1472	1073	797	2118	2284
**Particles picked**	177k	100k	500k	91k	111k	400k	550k
**Particles in good 2D classes**	-	47k	250k	71k	78k	270k	430k

2 Supplementary Figures

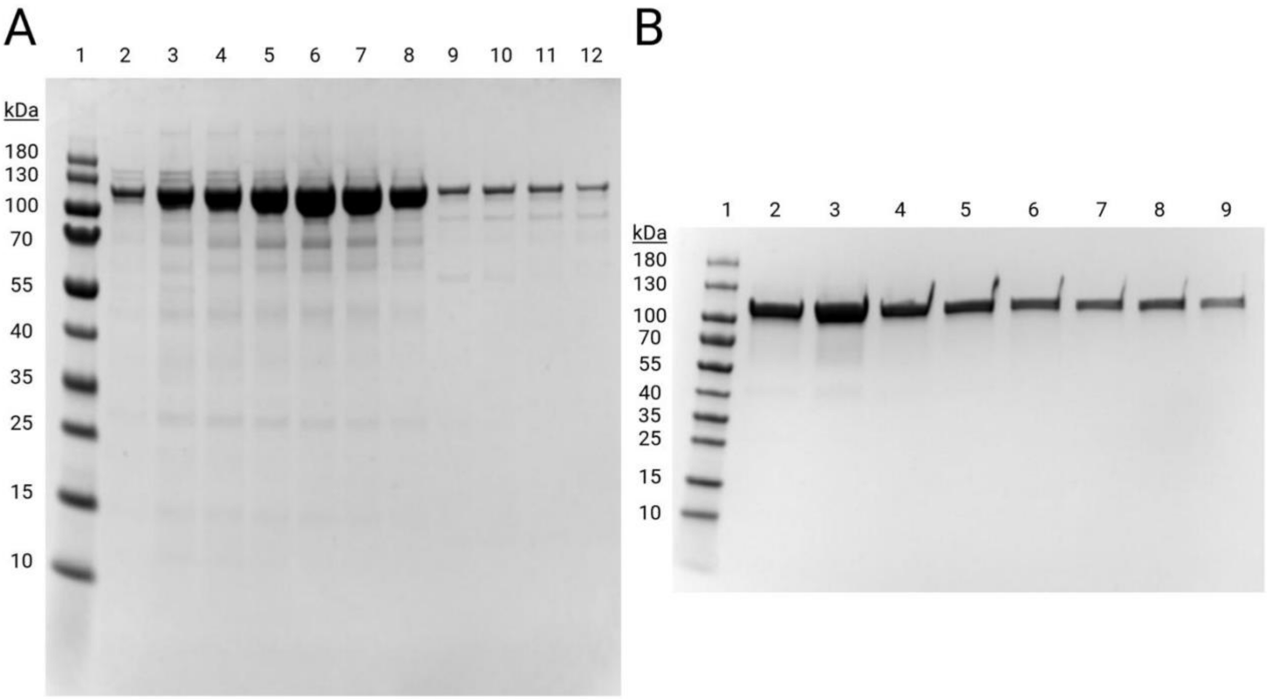

Supplementary Figure S1. SDS-PAGE of the standard and optimized purification methods. (A) SDS-PAGE gel corresponding to the SEC profile in Figure 1A. Lane 1 is the protein ladder, with molecular weights beside it. Lanes 2-4 correspond to peak 1. Lanes 5-9 correspond to peak 2. Lanes 9-12 correspond to peak 3. **(B)** SDS-PAGE gel corresponding to the SEC profile of the optimized method in Figure 3A. Lane 1 is the protein ladder, with molecular weights beside it. Lanes 2-9 correspond to fractions A10-B5, including fraction B3, respectively.
